# Screening of Human Circular RNAs as Biomarkers for Early Onset Detection of Alzheimer’s Disease

**DOI:** 10.3389/fnins.2022.878287

**Published:** 2022-07-05

**Authors:** Da Zheng, Rana Adnan Tahir, Yan Yan, Juan Zhao, Zhenzhen Quan, Guixia Kang, Ying Han, Hong Qing

**Affiliations:** ^1^Key Laboratory of Molecular Medicine and Biotherapy in the Ministry of Industry and Information Technology, Department of Biology, School of Life Sciences, Beijing Institute of Technology, Beijing, China; ^2^Key Lab of Universal Wireless Communications of Ministry of Education, Beijing University of Posts and Telecommunications, Beijing, China; ^3^Biomedical Engineering Institute, Hainan University, Haikou, China; ^4^Department of Neurology, Xuanwu Hospital of Capital Medical University, Beijing, China; ^5^Center of Alzheimer’s Disease, Beijing Institute for Brain Disorders, Beijing, China; ^6^National Clinical Research Center for Geriatric Disorders, Beijing, China

**Keywords:** Alzheimer’s disease, circular RNAs, miRNA, biomarker, bioinformatics, microarray analysis, gene ontology, circRNA-miRNA interactions

## Abstract

Circular RNAs (circRNAs) are a distinctive type of endogenous non-coding RNAs, and their regulatory roles in neurological disorders have received immense attention. CircRNAs significantly contribute to the regulation of gene expression and progression of neurodegenerative disorders including Alzheimer’s disease (AD). The current study aimed to identify circRNAs as prognostic and potential biomarkers in AD. The differentially expressed circRNAs among subjective cognitive decline, amnestic mild cognitive impairment, and age-matched normal donors were determined through Arraystar Human circRNA Array V2 analysis. The annotations of circRNAs-microRNA interactions were predicted by employing Arraystar’s homemade microRNAs (miRNA) target prediction tool. Bioinformatics analyses comprising gene ontology enrichment, KEGG pathway, and network analysis were conducted. Microarray analysis revealed the 33 upregulated and 11 downregulated differentially expressed circRNAs (FC ≥ 1.5 and *p*-values ≤ 0.05). The top 10 differentially expressed upregulated and downregulated circRNAs have been chosen for further expression validation through quantitative real-time PCR and subsequently, hsa-circRNA_001481 and hsa_circRNA_000479 were confirmed experimentally. Bioinformatics analyses determined the circRNA-miRNA-mRNA interactions and microRNA response elements to inhibit the expression of miRNAs and mRNA targets. Gene ontology enrichment and KEGG pathways analysis revealed the functional clustering of target mRNAs suggesting the functional verification of these two promising circRNAs. It is concluded that human circRNA_001481 and circRNA_000479 could be utilized as potential biomarkers for the early onset detection of AD and the development of effective therapeutics.

## Introduction

Circular RNAs (circRNAs) are a peculiar group of long, non-coding endogenous RNAs characterized by the existence of covalently closed RNA loops (Salzman et al., 2012, 2013; Jeck and Sharpless, 2014). These transcripts arise through the direct back-splicing and exon skipping of precursor RNAs (Starke et al., 2015; Chen, 2016). They lack free 5′ and 3′ ends and form a circular structure which makes them highly stable and resistant to exonuclease degradation (Wang and Wang, 2015; Chen, 2016).

Stability in expression and degradation resistance permit circRNAs in the application and development of novel clinically diagnostic biomarkers. CircRNAs also play a vital role in various diseases *via* competitively binding to the disease-related microRNAs (miRNAs; Zhang et al., 2017; Liu et al., 2018). The developmental phase and tissue-specific expressions of circRNAs signify their regulatory functions in gene expression (Wang et al., 2018). CircRNAs are widely found in eukaryotic cells and regulate gene expression *via* sponging particular miRNAs and consequently modulating their suppressive effect on RNA translation (Barrett and Salzman, 2016; Abdelmohsen et al., 2017; Han et al., 2017).

The precise understanding of circRNAs in the central nervous system (CNS) is hampered due to the lack of molecular tools required for the detection, quantification, and evaluation of circRNAs in physiologic processes and diseases such as Alzheimer’s disease (AD; Lukiw, 2013). CircRNA, ciRS-7, has emerged as a sponge of miR-7 and recently identified in promoting the degradation of BACE1 and APP in an NF-κB-dependent manner (Shi et al., 2017). Recent studies suggest that ciRS-7 could be useful as a diagnostic biomarker of AD and still further experiments are needed to reveal the functions of circRNAs contributing to AD pathology (Idda et al., 2018). The ability to freely cross the blood-brain barrier makes these circRNAs potentially invasive biomarkers for CNS disorders (Lu and Xu, 2016).

Alzheimer’s disease is the most common and prevalent form of dementia and one of the increasing economic and medical problems of the modern world (Alzheimer’s Association, 2019). It is characterized by the irreversible degeneration of cognitive functions, thinking, behavioral, and learning abilities, and is ranked as the sixth leading cause of death in the United States (Heron, 2018). It is estimated that at least 50 million people are living with AD or other dementias globally and it could surpass 152 million by 2050 in the absence of effective therapies (Patterson, 2018). Research studies have demonstrated that AD has a slow and progressive decline in cognitive functions over several years to decades and is categorized into three main stages: subjective cognitive decline (SCD), amnestic mild cognitive impairment (aMCI), and AD (Jiang et al., 2018). SCD is described as the transitional phase in the progression of AD pathology and also an early symptomatic expression of preclinical AD (Jessen et al., 2014). The research on SCD mainly focuses to identify the specific biomarkers of AD and also verifying SCD as a risk condition for MCI or AD (Schultz et al., 2015; Snitz et al., 2015; Sun et al., 2016; Chen et al., 2019). The dynamical model of cognitive decline demonstrates a subtle cognitive decline in SCD but within the standard cognitive performance range. The further cognitive decline leads to the aMCI-AD stage and still performance declines linearly (Jessen et al., 2014). The MCI phase has been recognized as an impairment of memory or cognition domains on a standard assessment and clinical cognitive staging in the absence of biomarkers (Jack et al., 2018). Still, extensive studies are needed to determine the dynamic model of cognitive decline in various stages for a better understanding of the quantitative mechanism of AD onset and progression (Jiang et al., 2018).

The current study aimed to identify the circRNAs as diagnostic biomarkers for the early onset detection of AD through circRNA microarray profiling followed by bioinformatics analyses. Here, the results revealed that hsa_circRNA_001481 and hsa_circRNA_000479 are significantly upregulated in the blood samples at various stages of AD patients. Gene ontology enrichment, KEGG pathway, and interaction network were constructed and analyzed for these upregulated human circRNAs to reveal the gene targets. Expression profiles of human circRNAs may lead to a better understanding of molecular insights and potential mechanisms for developing diagnostic markers and therapeutic methods.

## Materials and Methods

### Data Collection (Patients and Specimens)

Experiments of the current study were undertaken by the Ethics committee of Beijing Institute of Technology (BIT), Beijing, and all the samples were collected from the Xuanwu Hospital Capital Medical University, Beijing, with the consent of each subject. Blood specimen collection was divided into two phases: (i) diagnosis phase and (ii) validation phase. The first phase involved the collection of fresh blood samples from 55 participants (male: 26 and female 29). Three types of samples comprising SCD (22), aMCI (11), and AD (5) patient samples were collected along with normal controls (17) to determine the differentially expressed circRNAs. All the patients were diagnosed with transitional stages of cognitive decline through the consensus of two consultant psychiatrists based on the criteria for SCD, aMCI, and AD. It has been observed that approximately 30% of SCD patients had the habit of smoking and drinking along with hypertension. Some of the other patients having aMCI and AD also showed smoking, drinking, and a history of hypertension. Three SCD patients also had diabetes for the last 1, 3, and 12 years individually. No patient was suffering from Hepatitis or any other infectious diseases; however, heart diseases including blood pressure issues were reported in almost 13 patients. The six samples having different medical histories were selected for further circRNA microarray analyses. The second phase involves the collection of samples for the cross-validation of microarray analysis through qRT-PCR. The blood samples comprising SCD (3), aMCI (2), and AD (3) were collected in this phase. The SCD patient at the age of 70 years with no habit of smoking, drinking, or any other infectious disease along with the patient (65 years) with only a smoking habit, while third patient (76 years) suffering from heart disease and hypertension were utilized for the validation of differentially expressed circRNAs and to identify the uniform relative expression among all SCD patients. The aMCI patient with 7 years of hypertension and heart disease with an occasionally drinking habit was used for sample collection. The other aMCI with drinking and smoking habits and hypertension was also utilized for cross-validation of circRNAs. The AD samples with no habits of smoking and drinking and with hypertension and heart disease were also utilized in this phase. The initially collected samples were utilized for the high-throughput microarray sequencing analyses followed by the initial validation of differentially expressed circRNAs through qRT-PCR. The second phase involves the collection of samples for the cross-validation of differentially expressed circRNAs and to identify the potential and coverage of circRNAs among similar transitional stages of AD samples.

### Total RNA Extraction and Quality Control

Total RNA was extracted from the blood samples using TRIzol reagent (Invitrogen, NY, United States) according to the manufacturer’s instructions. The concentrations were measured through OD260 by employing NanoDrop ND-1000. Denaturing agarose gel electrophoresis was utilized to evaluate the RNA purity and gDNA contamination testing.

### RNA Labeling and Hybridization

Sample preparations, labeling, and array hybridization were executed under the company’s standard procedures (Arraystar Inc., MD, United States). Ribonuclease R (Epicentre, Inc.) digested the total RNAs and hence removed the linear RNAs which led to the enrichment of circRNAs. A random priming method (Arraystar Super RNA Labeling Kit; Arraystar) was used to amplify and transcribe the enriched circRNAs into fluorescent cRNA. The purification of labeled cRNAs was conducted *via* the RNeasy Mini Kit (Qiagen).

NanoDrop ND-1000 was utilized to determine the precise activity and concentration of labeled cRNAs (pmol Cy3/μg cRNA). A 1 μg of each labeled cRNA was fragmented by adding 1 μl of 25 × Fragmentation Buffer and 5 μl of 10 × Blocking Agent followed by the heating of the mixture for 30 min at 60^°^C. Subsequently, 25 μl of 2 × Hybridization buffer was used to dilute the labeled cRNA. A total of 50 μl of hybridization solution was dispensed into the gasket slide and assembled to create the circRNA expression microarray slide. The incubation of slides was carried out at 65^°^C for 17 h in an Agilent Hybridization Oven. Agilent Scanner G2505C was used to wash, fix, and scan the hybridized arrays.

### Circular RNAs Microarray Analysis

An Agilent feature extraction tool was employed to extract the raw data from the scanned images. The limma package of R language/software was used for quantile normalization and further data processing of raw data. Subsequently, low-intensity filtering was done to scrutinize the circRNAs from samples. The circRNAs that exhibited the “P” or “M” flags (“All Targets Value”) in at least 1 out of 6 samples were kept for further differential expression analyses. Profile differences (disease versus control) and “fold change” between the groups for each circRNA were computed and its statistical significance was assessed by *t*-test. Differentially expressed circRNAs having *p*-values < 0.05 and fold change > 1.5 were retrieved from microarray expression profiling.

### Annotation for Circular RNAs-MicroRNAs Interaction

Arraystar’s homemade miRNA target prediction software was used to generate the circRNAs-microRNA interactions and annotations of all the differentially expressed circRNAs by assessing the miRanda (Enright et al., 2003) and TargetScan (Lewis et al., 2005) databases. miRNA response elements (MREs) were hunted by utilizing the Arraystar software and the top five putative target miRNAs were selected based on seed match sequences.

### Quantitative Real-Time PCR

Quantitative real-time PCR (qRT-PCR) was performed to validate the overexpression of circRNAs, obtained from the microarray expression profiling. The top 10 circRNAs sorted on their fold change values were selected for qRT-PCR. The details of selected circRNAs including names, fold change values, *p*-values, chromosomal location, best transcript, gene symbol, and their up- or downregulation are mentioned in Table 2.

**TABLE 1:** Clinical details of specimens for circRNA microarray analysis.

Sr #	Clinical diagnosis	No.	Gender	Age	Medical history
1	NC	276	♀	69	
2		281	♂	62	
3	SCD	287	♀	61	12 years of Diabetes and 4 years of Hypertension
4		288	♀	75	History of Hypertension
5		275	♂	72	No Smoking, Drinking, Hypertension, or Heart disease
6	aMCI	274	♂	75	High Blood Pressure and Drinking

**TABLE 2:** Top 10 (upregulated and downregulated) differentially expressed circRNAs ranked by fold change.

CircRNA	FC	*P*-value	Chr.	Best transcript	Gene symbol	Regulation
hsa_circRNA_001481	3.6248831	0.02314	Chr5	NM_198449	EMB	UP
hsa_circRNA_016545	3.0679614	0.03505	Chr1	NM_001748	CAPN2	UP
hsa_circRNA_101543	2.4627691	0.00618	Chr15	NM_017684	VPS13C	UP
hsa_circRNA_100141	2.0285053	0.02437	Chr1	NM_080391	PTP4A2	UP
hsa_circRNA_078353	2.0063117	0.00326	chr6	NM_014892	SCAF8	UP
hsa_circRNA_104062	1.9204745	0.04166	Chr6	NM_005493	RANBP9	UP
hsa_circRNA_100978	1.9727913	0.02646	Chr11	NM_152715	TBCEL	UP
hsa_circRNA_000479	1.9500463	0.03432	Chr13	NM_033255	EPSTI1	UP
hsa_circRNA_003601	1.8470151	0.02749	Chr1	NM_018056	TMEM39B	Down
hsa_circRNA_042200	1.7439849	0.00482	Chr17	NR_027160	LRRC75A-AS1	Down

Total RNA was extracted from the blood samples and subsequently, reverse transcription was done to produce cDNA according to the standard protocols. SYBR green assay was utilized in qPCR to evaluate the expression levels of circRNAs. Divergent primers were designed and optimized to amplify the circular transcripts. CircRNA spliced sequences were retrieved from the database “circBase” to design the primers from Primer3 and Primer-Blast. Primers were synthesized from Sangon Biotech (Beijing, China) for qPCR. GAPDH was used as a reference to determine the relative expression of circRNAs.

### Bioinformatics Analyses

Bioinformatics analyses were carried out on differentially expressed circRNAs to predict the circRNA-miRNA-gene interactions and construct the network for the identification of gene targets that may be regulated by these selected circRNAs.

The miRDB database (Chen and Wang, 2020) was utilized to predict the miRNA interactions with gene targets and top upregulated circRNAs interactions with miRNAs and gene targets were mapped to an interaction network. CircRNA-miRNA-gene interactions network was constructed by using the Cytoscape software (Shannon et al., 2003). Gene ontology enrichment analysis was conducted to construct the annotations of the genes by using DAVID (Sherman and Lempicki, 2009). Gene functions comprising cellular components, physiological processes, and molecular functions were determined. KEGG pathway analysis was also performed to determine the involvement of genes in different physiological processes. Significant enrichment scores were examined and the involvement of genes in neurological disorders and their upregulations in brain tissues were observed.

### Cell Culture and Plasmid Construction

The human embryonic kidney cell lines (HEK293T) were purchased from American Type Culture Collection (ATCC, Manassas, VA, United States). The cells were cultured and maintained in Dulbecco’s modified Eagle’s medium containing 10% fetal bovine serum (GIBCO BRL, NY, United States) and 0.1% penicillin/streptomycin (Gibco, United States). The cultured cells were incubated in a humidified 5% CO_2_ atmosphere at 37^°^C.

The sequences of hsa_circRNA_001481 and hsa_circRNA_000479 were amplified through PCR and subsequently cloned into the pcDNA3.1 vector. The final constructs were validated by direct sequencing from Sangon Biotech (Shanghai, China).

### Transfection and Luciferase Assay

HEK293T cells were transfected at about 80% confluence with corresponding plasmid constructs and subsequently co-transfected with miRNAs by using a transfection reagent (Lipofectamine 2000, Invitrogen) as per the manufacturer’s recommendations. The miRNAs mimics of hsa_circRNA_001481 and hsa_circRNA_000479 were synthesized through Sangon Biotech, which is mentioned in Table 3.

**TABLE 3:** Top five miRNA binding sites of top differentially expressed circRNAs.

CircRNAs	MRE1	MRE2	MRE3	MRE4	MRE5
hsa_circRNA_001481	hsa-miR-1252-5p	hsa-miR-4644	hsa-miR-548m	hsa-miR-6758-5p	hsa-miR-6797-5p
hsa_circRNA_016545	hsa-miR-5193	hsa-miR-4685-5p	hsa-miR-339-5p	hsa-miR-6836-5p	hsa-miR-4638-5p
hsa_circRNA_101543	hsa-miR-643	hsa-miR-337-3p	hsa-miR-100-3p	hsa-miR-632	hsa-miR-19b-2-5p
hsa_circRNA_100141	hsa-miR-217	hsa-miR-574-5p	hsa-miR-595	hsa-miR-1323	hsa-miR-203a-3p
hsa_circRNA_078353	hsa-miR-6787-5p	hsa-miR-3960	hsa-miR-8072	hsa-miR-3141	hsa-miR-149-3p
hsa_circRNA_104062	hsa-miR-376c-5p	hsa-miR-376b-5p	hsa-miR-150-5p	hsa-miR-891a-3p	hsa-miR-135b-5p
hsa_circRNA_100978	hsa-miR-651-3p	hsa-miR-298	hsa-miR-154-5p	hsa-miR-335-3p	hsa-miR-424-5p
hsa_circRNA_000479	hsa-miR-942-5p	hsa-miR-4753-3p	hsa-miR-6873-3p	hsa-miR-6739-3p	hsa-miR-6809-3p
hsa_circRNA_003601	hsa-miR-214-3p	hsa-miR-3619-5p	hsa-miR-761	hsa-miR-634	hsa-miR-1226-3p
hsa_circRNA_042200	hsa-miR-612	hsa-miR-661	hsa-miR-6860	hsa-miR-6774-5p	hsa-miR-1285-3p

The cells were seeded into 24-well culture plates in which each group contains the three biological replicates. The cells were categorized into two basic groups: the negative control (NC) group involving the transfection with pcDNA3.1 and the mimics group comprising cells transfected with all miRNAs of both circRNAs. The pGL3 luciferase reporter vector was also used in the luciferase screening assay in addition to two NCs comprising miRNAs controls and luciferase reporters with or without circRNA_001481 and circRNA_000479. The cells were harvested after 48 h of transfection and then the relative luciferase activity of each miRNA was determined through the Dual-Luciferase Reporter Assay System. This experiment was repeated independently three times to increase the reproducibility and yield reliable and accurate results.

### Statistical Analysis

All the experiments were performed in triplicates and subsequent statistical analyses were performed to analyze and calculate the significance level of the data. Experimental results were expressed as mean ± standard error (SE), while group comparisons were tested through one-way ANOVA having less than 0.05 significant *p*-value. Pearson’s correlation analysis was carried out to analyze the relevance of expression. GraphPad Prism 8 (GraphPad Software, 2019) and Microsoft Excel were employed to analyze the experimental data and plot the graphs.

## Results and Discussion

Present work focused to determine the differentially expressed human circRNAs susceptible to AD patients for the early onset detection of disease through the circRNA as a biomarker *via* microarray analysis profiling and bioinformatics analyses.

The initial diagnosis phase involved the collection of 55 blood samples comprising 22 SCD, 11 aMCI, and 5 AD patient samples along with 17 normal controls to determine the differentially expressed circRNAs. SCD is referred to as the initial manifestation of the AD continuum which is the subjective experience of worsening cognitive performance (Jessen et al., 2020). The six samples including 3 SCD, 1 aMCI, and 2 NC were selected for circRNA microarray analysis. The details of these selected samples are mentioned in Table 1 along with their medical history. The medical history of these nominated donors shows the absence of smoking, coronary heart diseases, hepatitis, and other infectious diseases. SCD (#287) had diabetes for the last 12 years along with hypertension for 4 years while the SCD patient (#288) only had a history of specific hypertension. Participant #274 (aMCI) was suffering from a high blood pressure of around 170/90 mm Hg and later it was reduced to 140/80 mm Hg by regular medications. He also has a drinking addiction and has almost drank approximately 50 mL of liquor daily for the last 7 to 8 years.

### General Microarray Profiles

The differential expression of circRNAs was measured through human circRNA microarray analysis which detected the upregulation and downregulation of circRNAs among SCD, aMCI, and NC samples. The expression of circRNAs was compared between each group and approximately 6,000 to 8,000 differentially expressed circRNAs are detected in each comparison where significant ones were scrutinized based on fold change (≥1.5) and *p*-values (≤0.05). A total of 33 differentially expressed upregulated and 11 downregulated circRNAs were detected in the test versus control group. The comparison between aMCI versus control revealed 926 differentially expressed upregulated and 854 downregulated circRNAs, while aMCI versus test identified 1,300 upregulated and 1,169 downregulated circRNAs (Supplementary File 1).

**FIGURE 1:**
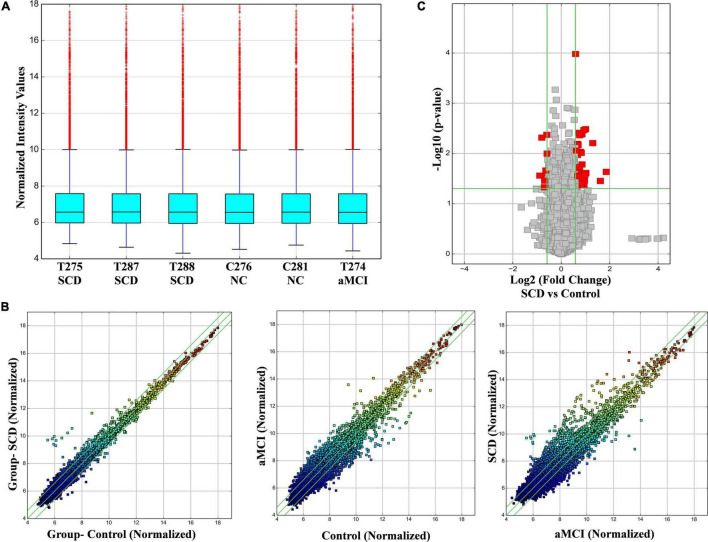
Microarray expression analysis of human circRNAs in SCD and aMCI against control samples. **(A)** Box plot presenting the normalized intensity values of each sample which are almost the same in each sample. **(B)** The scatter plots represent the variability of expression between each sample. The values of the *X* and *Y* axes in the scatter plot are the normalized signal values of the samples (log2 scaled). The green lines are fold change lines. The CircRNAs above the top green line and below the bottom green line indicated more than a 1.5-fold change of circRNAs between the two compared samples. **(C)** Volcano plot, showing the red points that represent the differentially expressed circRNAs with statistical significance. The vertical lines correspond to 1.5-fold up and down, respectively, and the horizontal line represents a *p*-value of 0.05.

The distribution of intensities from all the samples was visualized in the box plot which compares the distribution of expression values between each group after normalization (Figure 1A). The expression variability of circRNAs was assessed through the scatter plots between each sample group. The scatter plots between test versus control, control versus aMCI, and test versus aMCI were constructed accordant with the fold change (Figure 1B). A volcano plot was constructed to determine the differential expression between test versus control which helped to identify the altered circRNAs with statistical significance (Figure 1C). Fold change and *p*-value filtering were considered in the volcano plot, thereby allowing to separate the circRNAs with variability and significance.

**FIGURE 2:**
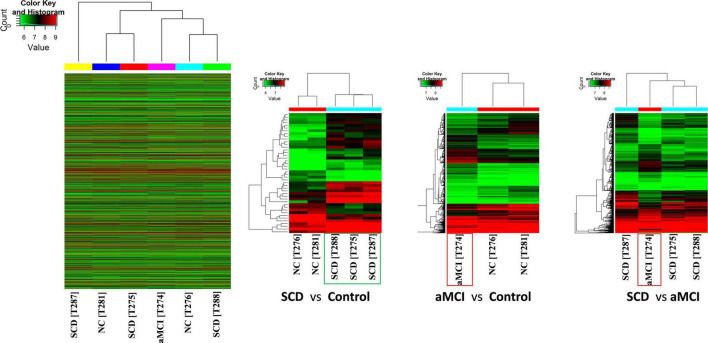
Hierarchical cluster analysis presents the expression profiles of each group. High relative expression is indicated by red color while low relative expression is in green color. Expression profiles of similar groups were clustered and represented through the dendrogram. Heat maps of each comparison show the higher relative expression of SCD samples as compared to controls. Control samples (C276 and C281) and test samples (SCD: T275, T287, and T288 and aMCI: T274).

Hierarchical clustering was used to determine the expression levels of samples. Cluster analysis placed the samples per their expression levels and compared each group (Figure 2). Heat maps were used to identify the expression levels of each sample and higher expression levels of test samples were observed as compared to control and aMCI samples. Groups having similar expression levels are clustered through the dendrogram.

Fold change filtering was applied to sort out the top upregulated and downregulated differentially expressed circRNAs and selected them for subsequent validation through RT-qPCR. The top circRNAs are mentioned in Table 2 along with annotations of circRNAs. A total of 30 circRNAs are exonic, while 2 are sense overlapping and only 1 lies in the intronic region.

The top potential binding target miRNAs of differentially expressed circRNAs were also predicted and considered as the potential binding targets as mentioned in Table 3.

### Validation of Circular RNAs by Real-Time-qPCR

**FIGURE 3:**
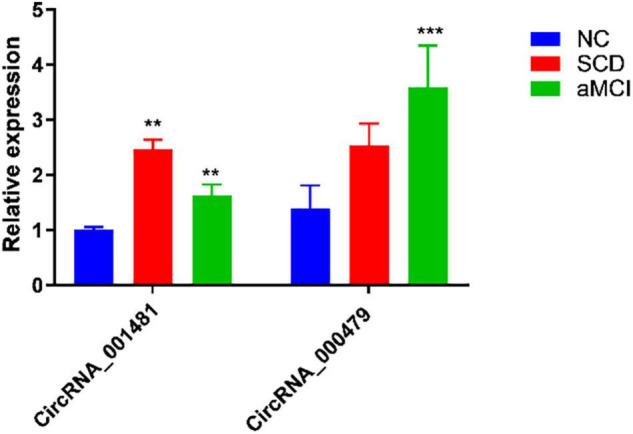
RT-qPCR analysis of hsa_circRNA_001481 and hsa_circRNA_000479. The relative expression of SCD and aMCI are significantly upregulated in hsa_circRNA_001481 as compared to NC. The relatively higher expression in aMCI is also observed in hsa_circRNA_000479. The values are the average values of three independent experiments (**P < 0.01, ***P < 0.001).

High-throughput microarray assay yielded the differentially expressed circRNAs which required verification through experimental techniques. Top altered circRNAs with relatively high fold change as mentioned in Table 2 are selected for verification through RT-qPCR. As compared with the reference, hsa_circRNA_001481 and hsa_circRNA_000479 were significantly upregulated in the test samples (Figure 3).

The hsa_circRNA_001481 has been identified as significantly upregulated in SCD and aMCI compared with control samples and further verified through RT-qPCR. Upregulated expressed circRNAs between control versus SCD revealed the highest fold change 3.62 of hsa_circRNA_001481, while 2.40 and 1.51 FC in control versus aMCI and SCD versus aMCI samples were identified, respectively. The hsa_circRNA_001481 is a sense-overlapping circRNA and its official gene symbol is EMB that is located on the negative strand of chromosome 5. The significant upregulation of hsa_circRNA_000479 in microarray analysis was also validated by RT-qPCR. The fold change 1.9 of this circRNA was observed in SCD versus control groups, while 2.2 was observed in the upregulation of SCD versus aMCI. This exonic circRNA is located on the negative strand of chromosome13 and its gene symbol is Epithelial stromal interaction 1 (EPSTI1).

The binding sites of these circRNAs are analyzed to mediate the interactions of miRNA with MREs and to determine the target interactions of circRNAs. The details of MREs of both hsa_circRNA_001481 and hsa_circRNA_000479 are provided in Supplementary File 2.

### Construction of Circular RNAs-MicroRNAs-mRNA Networks

**FIGURE 4:**
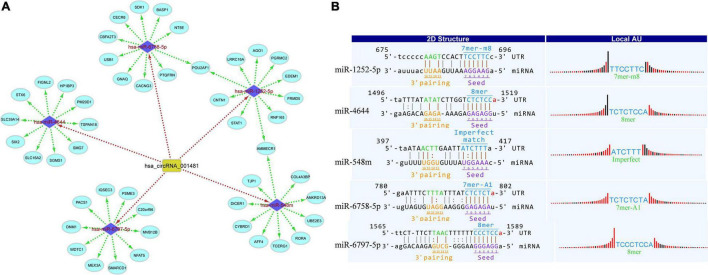
**(A)** CircRNA_001481_miRNA_mRNA interaction network comprising 5 miRNAs and 50 targets. The top 10 mRNA targets of each miRNA are shown in this network constructed by Cytoscape 3.6. **(B)** Top MREs of miRNAs and 2D structure showing the MRE sequence analysis for hsa_circRNA_001481.

Circular RNAs act as miRNA sponges that play significant roles in various diseases. Therefore, the interaction network of circRNAs-miRNA-mRNA was predicted and constructed to identify the potential functions of differentially expressed circRNAs. miRDB and miRWalk 3.0 (also utilizes miRTarBase, miRDB, and TargetScan databases) were used to predict the miRNA targets. The miRWalk 3.0 predicts the targets through the experimentally validated interactions and machine learning algorithm. The top target genes were selected for each miRNA for the construction of circRNAs-miRNA-mRNA interactions through the bioinformatics tools, that is, Cytoscape 3.6. The target interaction network of hsa_circRNA_001481 was constructed and presented in Figure 4A. The upregulated hsa_circRNA_001481 was predicted to inhibit the expression level of hsa-miR-1252-5p, hsa-miR-4644, hsa-miR-548m, hsa-miR-6758-5p, and hsa-miR-6797-5p and further promote the expression of target genes. POU2AF1 and AMMECR1 genes are found as the common targets between hsa-miR-1252-5p and hsa-miR-6758-5p, and between hsa-miR-1252-5p and hsa-miR-548m, respectively.

**FIGURE 5:**
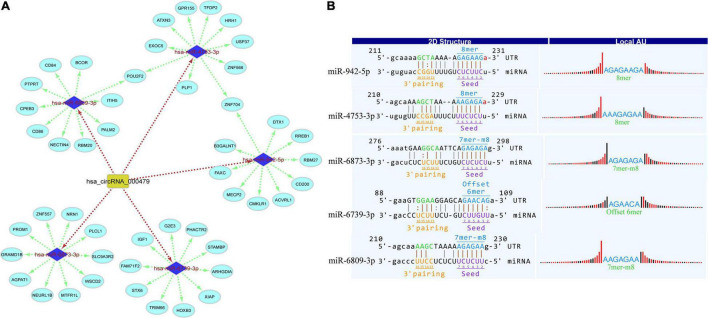
**(A)** CircRNA_000479_miRNA_mRNA interaction network constructed by Cytoscape. **(B)** Top MREs of hsa_circRNA_000479 are shown, predicted by Targetscan and miRanda databases.

The MREs of hsa_circRNA_001481 were predicted and shown in Figure 4B along with the sequence analysis. The 3′ pairing sequence of hsa_circRNA_001481, target miRNA seed type, and MRE sequence are illustrated in the 2D structure. The element matching perfectly based on their seeds are selected for each miRNA. The miR-548m exhibited imperfect pairing with their predicted MRE sequence. The hsa_circRNA_000479-miRNA_mRNA network was also constructed which contains 5 miRNAs and 50 targeted genes (Figure 5A). The initial-miR-4753-3p displayed the common targets ZNF704 and POU2F2 with hsa-miR-942-5p and hsa-miR-6809-3p, respectively. The upregulated hsa_circRNA_000479 was also predicted to increase the expression level of miRNA target genes through the inhibition of hsa-miR-942-5p, hsa-miR-4753-3p, hsa-miR-6873-3p, hsa-miR-6739-3p, and hsa-miR-6809-3p. The 2D MRE sequence analysis of hsa_circRNA_000479 identified the target miRNA seed type, 3′ pairing sequence for hsa_circRNAs_000479, and MRE sequence as depicted in Figure 5B.

### GO and Pathway Analysis of Target mRNAs

Both hsa_circRNA_001481 and hsa_circRNA_000479 may play key roles in the molecular mechanisms through the regulation of target mRNAs on the report of these interactions. These putative mRNAs were further investigated in GO, KEGG pathway, and tissue expression analyses for the functional characterization of these circRNAs.

**FIGURE 6:**
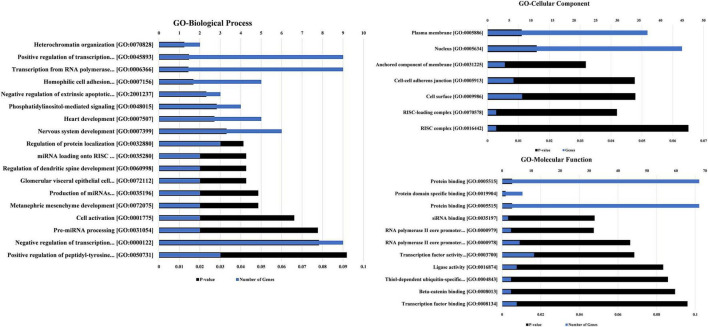
The GO annotations for biological process, molecular function, and cellular component of the predicted mRNAs regulated by both scrutinized circRNAs. The GO annotations along with the number of genes and *P*-value are also mentioned in the chart. The lowest *p*-value is utilized for sorting the GO terms in the figure.

The biological process, molecular function, and cellular component were predicted and analyzed in GO bioinformatics analyses (Figure 6). In terms of biological process, nervous system development, positive and negative regulations of transcription, and transcription from RNA polymerase II were identified as significant terms that describe a series of biological activities. Protein binding and transcription factor activity are the meaningful enrichment terms in the category of molecular function showing the functional role at the molecular level. GO Cellular component determined the nucleus and plasma membrane as significant terms elucidating the components of a cell.

**FIGURE 7:**
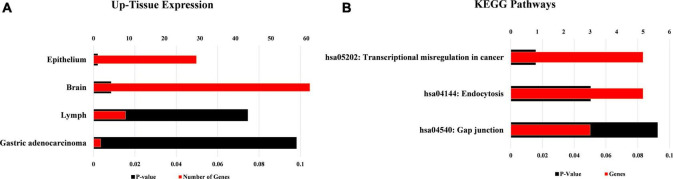
Functional annotations of target mRNAs. **(A)** Up-tissue expression of target mRNAs regulated by these both circRNAs. **(B)** KEGG pathway analysis of these targets’ mRNAs.

The tissue expression analysis was conducted to determine the upregulation of target mRNAs at the tissue level (Figure 7A). The upregulations were observed in the brain, epithelium, lymph, and gastric adenocarcinoma in terms of tissue expression. The significant upregulation of these mRNAs was observed in brain tissues which accounts for almost 60% of the total genes providing evidence for the functional verification of hsa_circRNA_001481 and hsa_circRNA_000479. The KEGG pathway analysis exhibited the top three pathways’ transcriptional misregulation in cancer, endocytosis, and gap junction (Figure 7B). Pathway analysis suggests that these genes may play a significant role in multiple metabolic events.

**FIGURE 8:**
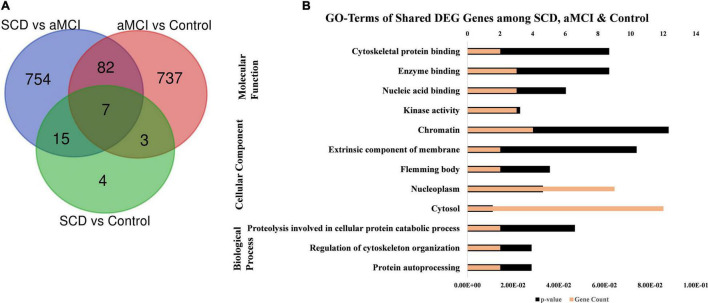
GO annotations of upregulated DEGs among SCD, aMCI, and control groups. **(A)** Venn diagram showing the common upregulated DEG among SCD versus aMCI, aMCI versus control, and SCD versus control. **(B)** GO terms including biological process, molecular function, and cellular component of DEGs (25) from SCD versus control, also share with other SCD versus aMCI and aMCI versus control groups.

**FIGURE 9:**
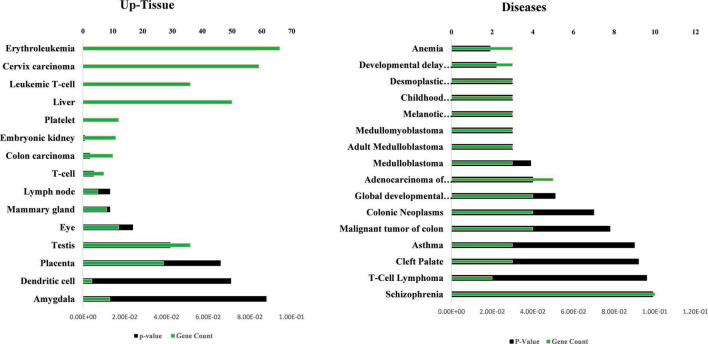
Up-tissue expression and involvement in diseases of all the mutual DEGs (107) between SCD versus aMCI, aMCI versus control, and SCD versus control. The annotation terms are mentioned with a number of genes along with significance (*p*-value).

The GO, tissue expression, and disease analysis of differentially expressed genes (DEGs) from microarray analysis were also conducted to determine the biological functions, cellular component, molecular function, upregulated expression in tissues, and their involvement in diseases. The common DEG genes among upregulated SCD versus control, aMCI versus Control, and SCD versus aMCI were identified through the Venn diagram as represented in Figure 8A. The 25 genes from SCD versus Control also common with groups were utilized for their biological functions, biological process, and cellular component (Figure 8B). The second analysis involves all mutual DEGs between aMCI versus Control, SCD versus Control, and SCD versus aMCI, which was employed for their upregulation expression in tissues followed by disease analysis (Figure 9). It has been seen that the highest number of DEGs have been found in Schizophrenia in disease analysis.

### Circular RNAs Act as MicroRNAs Sponges

**FIGURE 10:**
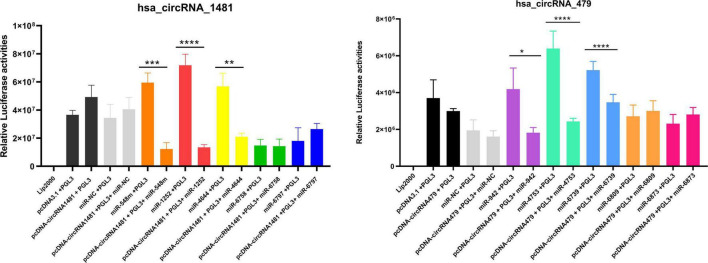
The relative luciferase activities of hsa_circRNA_1481 and hsa_circRNA_000479 showing the expression level of each miRNA. The relative luciferase activities were calculated in HEK293T cells co-transfected by five miRNA mimics are depicted in the bar graph (*P < 0.05, **P < 0.01, ***P < 0.001, and ****P < 0.0001). Experimental results are expressed as mean ± standard error.

It has been documented that cirRNAs act as miRNA sponges that competitively suppress and naturally sequester miRNA activity (Hansen et al., 2013). As presumed, CircRNAs can also function as a decoy to regulate the mRNA expression similarly. It has been proposed and investigated that either hsa_circRNA_001481 or hsa_circRNA_000479 function as competing endogenous RNAs to target miRNAs and suppress their expression. The miRNAs having binding sites at the 3′ UTR region of hsa_circRNA_001481 are hsa-miR-1252-5p, hsa-miR-4644, hsa-miR-548m, hsa-miR-6758-5p, and hsa-miR-6797-5p were determined through bioinformatics analysis as mentioned above. Similarly, hsa-miR-942-5p, hsa-miR-4753-3p, hsa-miR-6873-3p, hsa-miR-6739-3p, and hsa-miR-6809-3p were identified as the target binding miRNAs of hsa_circRNA_000479. Luciferase screening assay was conducted to verify corresponding miRNAs binding to hsa_circRNA_001481 and hsa_circRNA_000479. The miRNAs mimics were co-transfected with the luciferase reporter by utilizing HEK293T cells and determined the expression level with and without circRNAs. The relative luciferase activities were calculated and plotted on a graph through GraphPad Prism8 as shown in Figure 10. It has been seen that hsa_circRNA_001481 significantly reduced the expression of hsa-miR-548m (*P* < 0.0004), hsa-miR-1252-5p (*P* < 0.0008), and hsa-miR-4644 (*P* < 0.05) as compared to the controls. Luciferase activities indicate that hsa_circRNA_001481 may function as a sponge for hsa-miR-548m, hsa-miR-1252-5p, and hsa-miR-4644 to regulate the expression level of EMB. There were no convincing variations in the expression level of hsa-miR-6758-5p and hsa-miR-6797-5p that do not show any effect of hsa_circRNA_001481.

The relative luciferase activities of hsa_circRNA_000479 against five miRNAs mimics were calculated and it has been observed that hsa_circRNA_000479 reduced the luciferase reporter activities of hsa-miR-942-5p (*P* < 0.0455), hsa-miR-4753-3p (*P* < 0.0001), and hsa-miR-6739-3p (*P* < 0.0001) as compared with NCs. Luciferase screening suggests that hsa_circRNA_000479 may function as a sponge for hsa-miR-942-5p, hsa-miR-4753-3p, and hsa-miR-6739-3p to regulate the expression level of EPSTI1. However, the hsa_circRNA_000479 did not show the sponge properties for hsa-miR-6873-3p and hsa-miR-6809-3p, and hence no expression level differences were detected for both these miRNAs.

The aggregation and differential expression of circRNAs in neural tissues in the course of aging through neural genes have received immense attention in neurosciences (Ashwal-Fluss et al., 2014; Westholm et al., 2014). The highly expressed thousands of circRNAs in the mammalian brain have been reported in the advanced sequencing studies of dissected brain tissues and differentiated neuronal cell lines. These circRNAs are highly conserved between humans and rodents, developmentally regulated, and enriched in synaptic fractions in neurons (Rybak-Wolf et al., 2015; You et al., 2015).

A study by Rybak-Wolf et al. (2015) has identified a large number of upregulated circRNAs in neuronal differentiation and most of them originated from host genes, involved in neuronal functions. Furthermore, You et al. (2015) utilized an RNA *in situ* hybridization technique to validate the synaptic localization of circRNAs and found the enrichment of circRNAs with a majority of them derived from the genes that encode synaptic proteins in brain samples.

Many circRNAs have been reported and documented in neurological disorders including AD, schizophrenia, Parkinson’s disease, and multiple sclerosis (Lukiw, 2013). CircRNA study has become a research hotspot due to their significant roles in disease progression (Zhang et al., 2018) and systematic research is underway to reveal the functional roles of circRNAs and elaborate on the regulation patterns of the human transcriptome (Guo et al., 2014; Rybak-Wolf et al., 2015).

It has been clearly stated from numerous genome-wide surveys that the circRNAs normally arise during the transcription process rather than the circumstantial expression of this type of ncRNAs (Guo et al., 2014; Rybak-Wolf et al., 2015). The dynamic expression of circRNAs in the brain indicates that their accumulation and expression are age-dependent (Westholm et al., 2014). The resistance ability of circRNAs against RNase cleavage extends their stability which enables circRNAs as a preferable choice for molecular markers as compared to linear RNAs (Enuka et al., 2015). The dynamic expression of circRNAs and their transcriptomic analyses in neurodegenerative disorders needs to be performed and demonstrated effectively. The reduced expression of circRNA for miRNA-7 has been reported in AD brains while it comprises tandem antimiRNA-7 sequences (Lukiw, 2013).

In the current investigation, the expression profiles of circRNAs among SCD, aMCI, and AD were discovered to screen the circRNAs mainly through microarray analysis followed by the bioinformatics approaches. SCD is defined as the experience of worsening or more frequent confusion or memory loss. It is a form of cognitive impairment and one of the earliest noticeable symptoms of AD and related dementias. The objectives of the current study were also to identify the potential biomarkers for early onset detection of AD. The three samples of the main SCD group were taken for microarray analysis with a smaller number of samples from other groups. The reliability of differential expressed data is extensively verified through further experimental and computational analyses and techniques. The top 10 differentially expressed up- and downregulated circRNAs extracted from microarray analysis were further validated through qRT-PCR, and hsa-circRNA_001481 and hsa_circRNA_000479 were confirmed with a high degree of sensitivity and specificity. Therefore, the current study is not limited to samples but extensive analyses were carried out to validate the novel potential circRNAs as biomarkers for the early onset detection of AD. In future, the studies will be conducted with a greater number of samples to identify and analyze the novel potential circRNAs.

Our findings have indicated that hsa-circRNA_001481 was significantly upregulated in SCD and aMCI expression profile as compared to the healthy controls highlighting it as a potential biomarker for early stage AD patients.

The EMB gene harbors nine exons that encode an embigin protein having a 30-kDa molecular weight when unglycosylated and its chromosomal location is 5q11.1 (Ozawa et al., 1988). Embigin is involved in the formation of neuromuscular junctions, cell migration, and early embryonic development depending upon neural cell adhesion molecules (Zhou et al., 2020). Embigin upholds the catalytic activity of MCT2 for synergistic transfer of lactic acid between neurons and glial cells (Wilson et al., 2005), which plays a vital role in long-term procedural memory formation and brain energy metabolism (Pérez-Escuredo et al., 2016).

GWAS study showed the genome-wide significance of the EMB gene with mRNA expression level in cis genetic linkage with rs10940346 associated with schizophrenia (Li et al., 2017). EMB as a susceptible gene for schizophrenia has been confirmed and consistent with the GWAS results (Li et al., 2017; Pardiñas et al., 2018). The polymorphism at 3′-UTR of EMB is prominently linked with schizophrenia in the Chinese Han population but its effect on EMB expression still needs to be elucidated (Zhou et al., 2020).

The hsa_circRNA_000479 also exhibited a higher specificity and sensitivity that might be a non-invasive biomarker for early stage AD patient screening tests. EPSTI1, an interferon response gene was initially recognized in breast cancer that induces stromal fibroblast (Gudjonsson et al., 2003). Mo and Chae (2017) have described the possible role of EPSTI1 as a candidate gene for systematic lupus erythematosus and highlighted that variants at EPSTI1 could be utilized as potential genetic markers for systematic lupus erythematosus susceptibility.

Luo et al. (2016) have conducted a study to determine and compare the expression patterns of AD and vascular dementia (VaD) from peripheral blood samples. A set of signature genes were extracted in the experiment including EPSTI1, which might be the potential biomarkers for early detection of AD and VaD (Luo et al., 2016).

The circRNAs have the potential to be utilized as diagnostic and prognostic biomarkers for various complex disorders due to their high abundance in the human body and their excellent stability (Zhang et al., 2018). An average half-live of circRNAs is 48 h as compared to 10 h for mRNAs in the resistance to RNA endonucleases cleavage (Jeck and Sharpless, 2014). The circRNAs have been extensively studied to determine their potential as non-invasive biomarkers for human diseases but still, many complex mechanisms and functions remain elusive (Li et al., 2015; Memczak et al., 2015; Zhang et al., 2018). The circRNA transcripts are preferable molecules for the actual detection of neurological disorders due to their high abundance in peripheral blood and brain, compared to other tissues. Particularly, circRNA-based detection might be an appropriate approach for neurodegenerative diseases as disruptions of circRNAs-miRNA binding are reported in AD (Lukiw, 2013).

The miRNAs play a vital role in the regulation of gene expression through posttranscriptional regulation, hereby it gained much attention for a better understanding of miRNAs regulatory mechanisms. Recently, miRNA sponging has been identified as one of the key functions of circRNAs that leads to the inhibitory activity of miRNAs (Qi et al., 2015; Meng et al., 2017).

mRNAs and circRNAs utilize MREs for competitively binding to inadequately targeted miRNAs and construct the regulatory network of competing for endogenous RNA (Jeck and Sharpless, 2014). The translation process interrupts due to the binding of mRNA with miRNAs; however, circRNAs remain resistant and stable from the degradation by RNA exonucleases (Panda, 2018; Yu and Liu, 2019). CircRNAs may transport or store miRNAs momentarily, and modulate the expression of miRNA-associated target genes (Duk and Samsonova, 2021). Many studies have documented the significance of miRNAs in the elucidation of molecular pathology in different diseases (Misir et al., 2020a,b). CircRNAs as miRNA sponges and circRNAs-miRNAs-mRNAs networks may deliver a vital role in understanding and modulating the expression of miRNA-related genes in different diseases (Zhao et al., 2019).

The accumulation of circRNAs appears in the brain with time, and so their metabolism can be associated with healthy aging. Age-dependent accumulation of circRNA transcripts has been determined in Drosophila neural tissue (Westholm et al., 2014) and demonstrating that the function of circRNAs is a well-conserved genomic feature for brain physiology. The circRNA transcriptome is also widely reshaped through porcine and human brain development (Szabo et al., 2015; Venø et al., 2015). The fact that the expression patterns of circRNAs are affected during aging or neurodegeneration processes could be determined to identify and study the more promising circRNAs biomarkers for human diseases and their clinical implementation.

## Conclusion

The current study reported the preferentially expressed profiles of circRNAs from the blood samples susceptible to AD individuals. Human microarray analysis followed by bioinformatics approaches revealed the hsa_circRNA_001481 and hsa_circRNA_000479 as differentially expressed circRNAs that could be utilized for early diagnosis of AD. The precise expression and stability of numerous circRNAs enable them to model candidates as diagnostic tools in neurodegenerative disorders and aging. The circRNAs-based effective therapeutic approaches and diagnostic biomarkers could be developed for AD through the molecular insights of circRNAs in disease development. Collectively, circRNAs are novel promising and favorable biomarkers for human diseases owing to distinct functional and structural features. Recent advances in Biotechnology and Bioinformatics will assist to determine the increasingly circRNAs as potential biomarkers for clinical applications.

## Data Availability Statement

The original contributions presented in this study are included in the article/Supplementary Material, further inquiries can be directed to the corresponding authors.

## Ethics Statement

The studies involving human participants were reviewed and approved by Han Ying, Department of Neurology, Xuanwu Hospital of Capital Medical University, Beijing, China. The patients/participants provided their written informed consent to participate in this study.

## Author Contributions

HQ, YH, and ZQ conceived, designed, and supervised the experiments and manuscript. DZ, RT, and YY conducted the sampling and further lab experiments. RT performed the computational analyses and drafted the manuscript. All authors contributed to the article and approved the submitted version.

## Conflict of Interest

The authors declare that the research was conducted in the absence of any commercial or financial relationships that could be construed as a potential conflict of interest.

## Publisher’s Note

All claims expressed in this article are solely those of the authors and do not necessarily represent those of their affiliated organizations, or those of the publisher, the editors and the reviewers. Any product that may be evaluated in this article, or claim that may be made by its manufacturer, is not guaranteed or endorsed by the publisher.
