# Supplementary material for: Screening of Human Circular RNAs as Biomarkers for Early Onset Detection of Alzheimer’s Disease
Source: Front Neurosci. 2022 Jul 5;16:878287. doi: 10.3389/fnins.2022.878287 (PMC9296062; doi:10.3389/fnins.2022.878287)
Supplement: Supplementary file 1 [file Image_1.pdf]

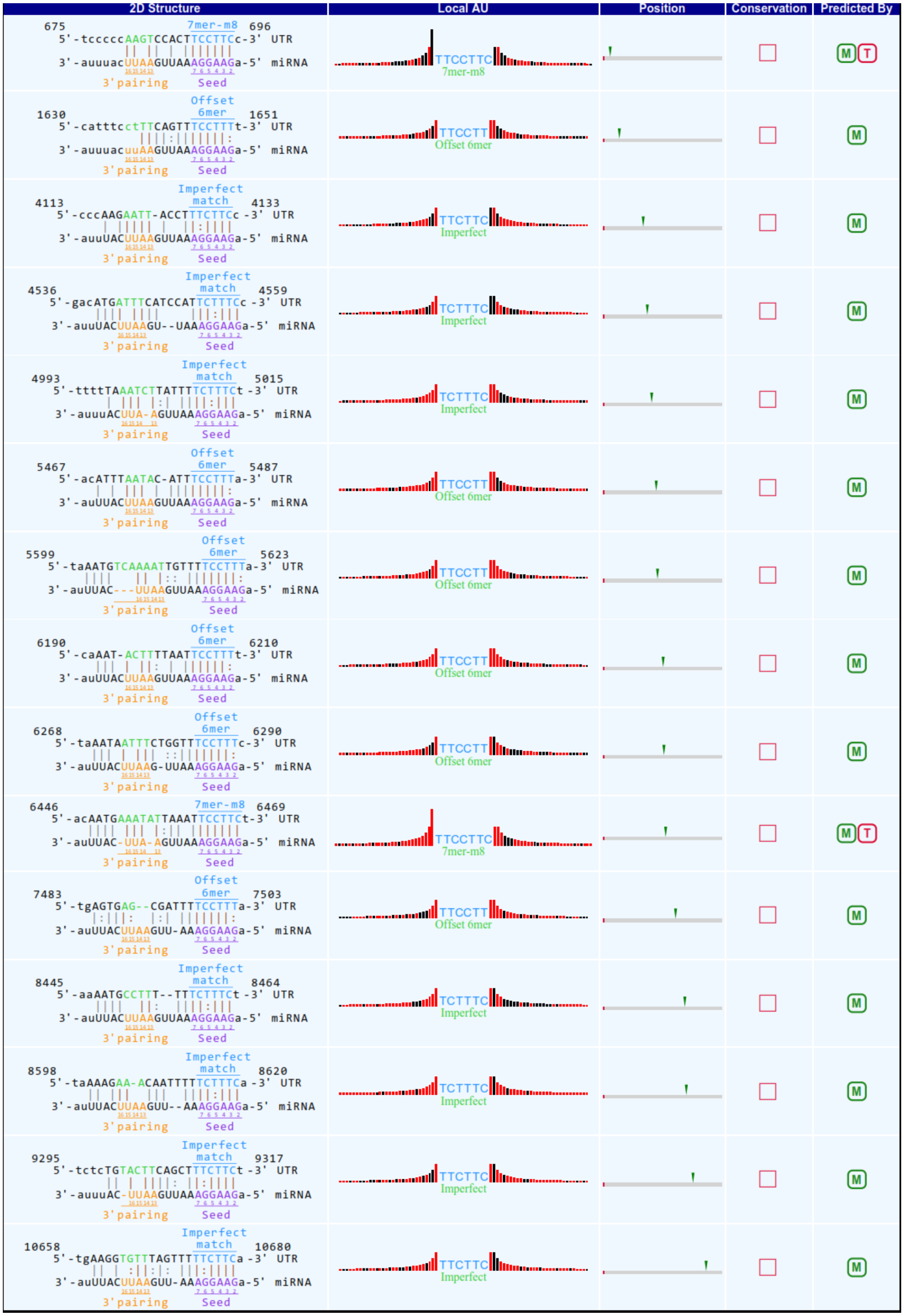

Figure 1: The binding sites of hsa_circRNA_001481 with hsa-miR-1252-5p, are shown in 2D structure interactions

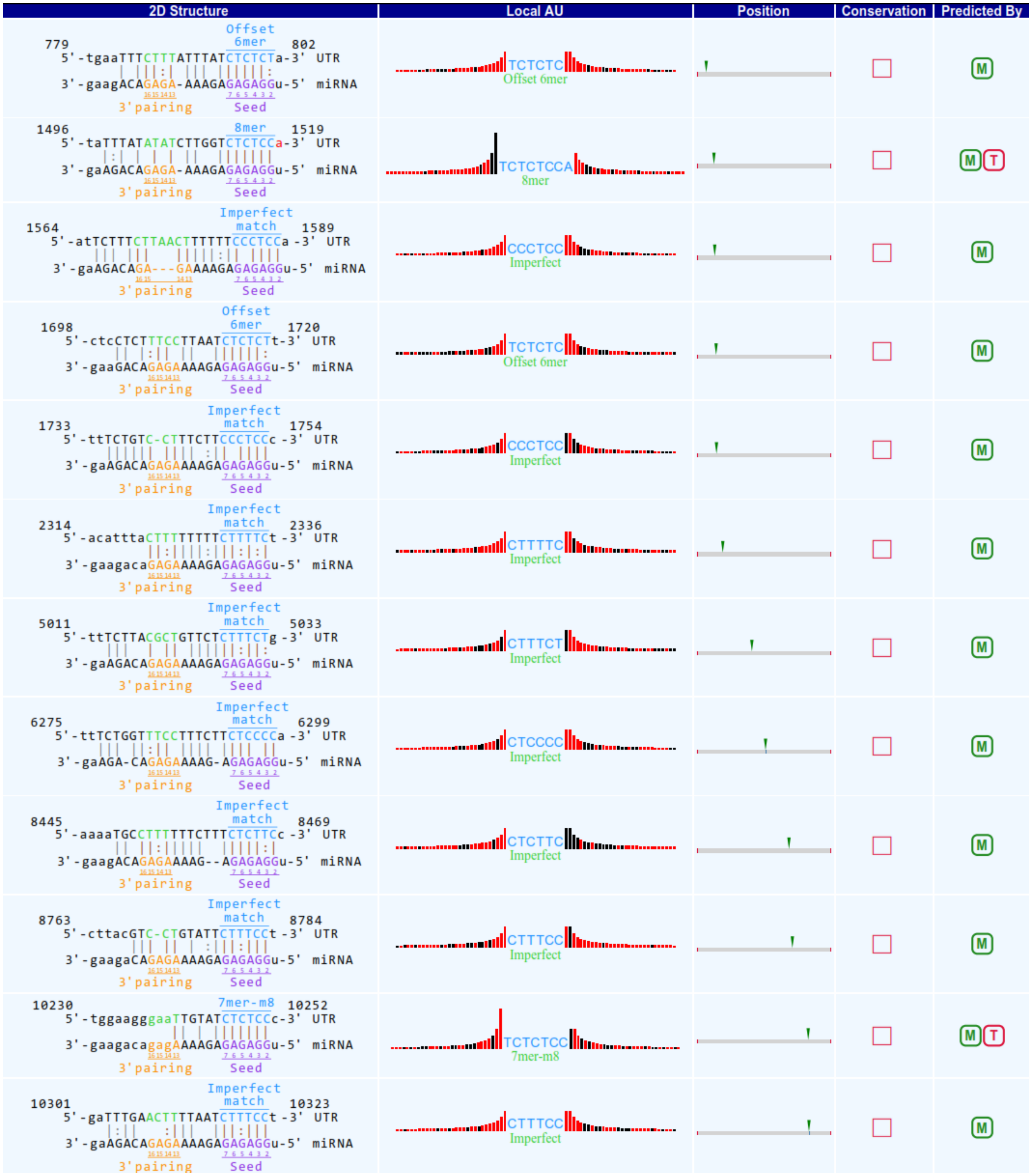

Figure 2: The binding sites of hsa_circRNA_001481 with hsa-miR-4644 along with predicted tool and Local AU

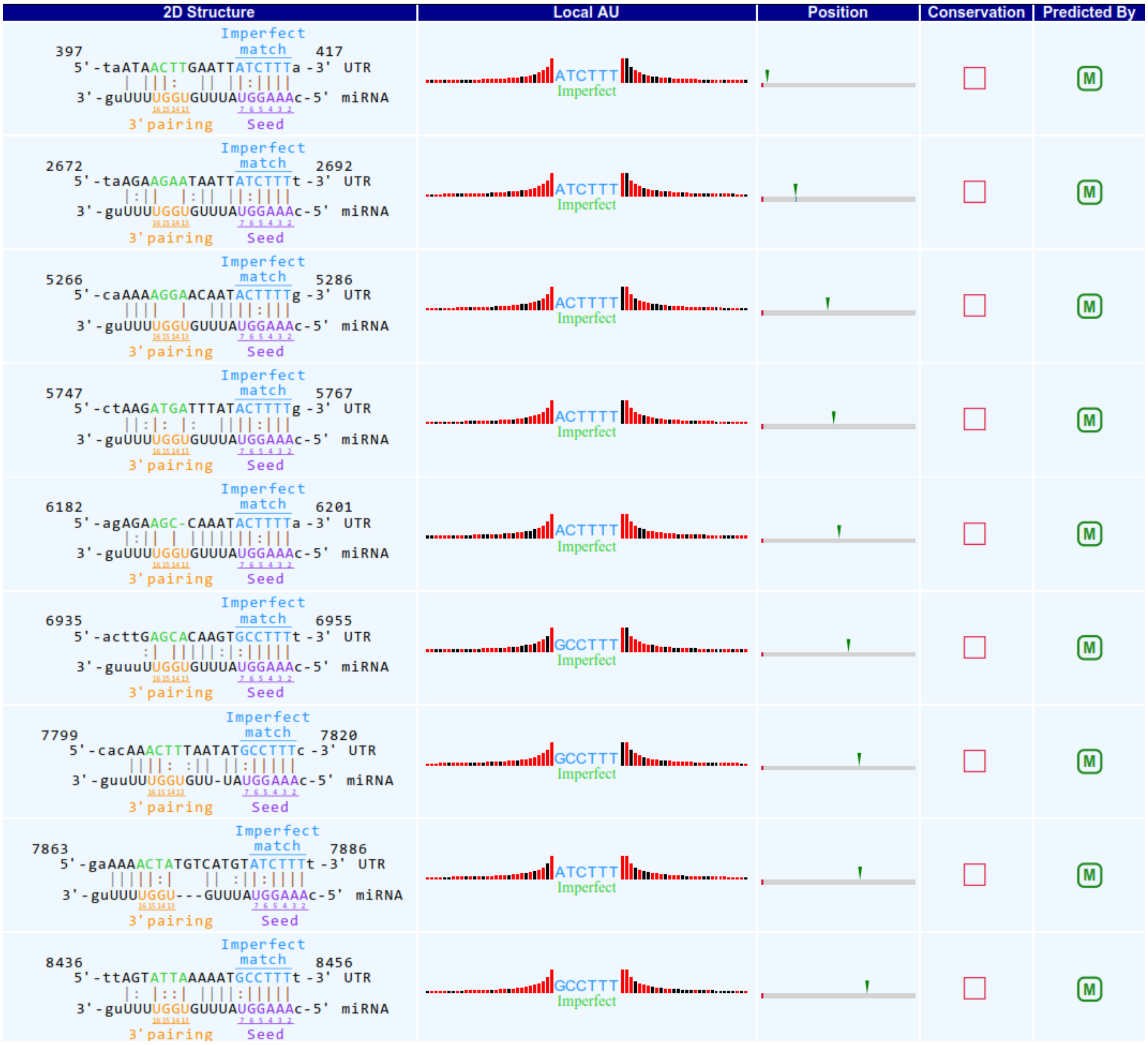

Figure 3: The binding sites of hsa_circRNA_001481 with hsa-miR-548m are presented.

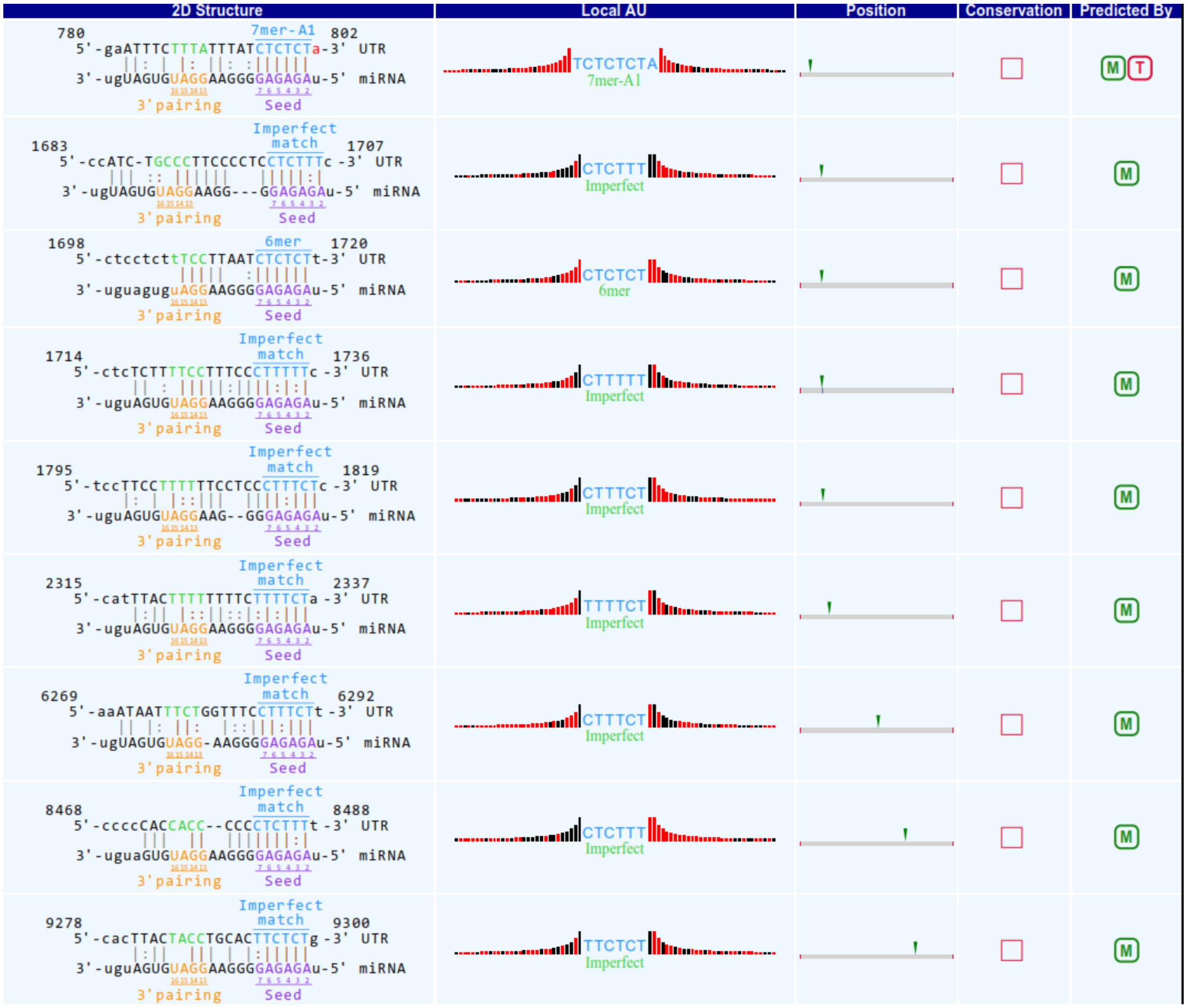

Figure 4: The binding sites of hsa_circRNA_001481 with miRNA (hsa-miR-6758-5p) response elements (MRE)

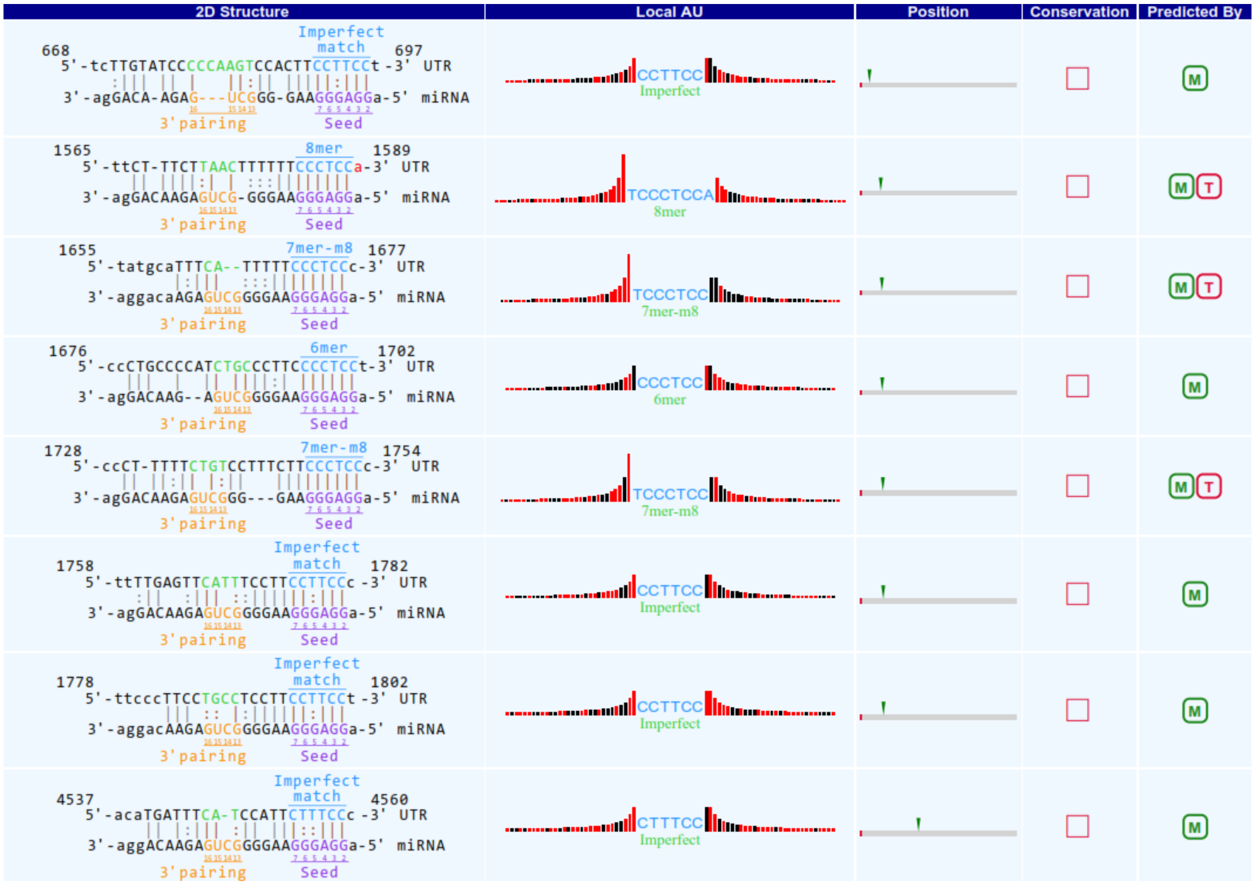

Figure 5: The binding sites of hsa_circRNA_001481 with hsa-miR-6797-5p response elements

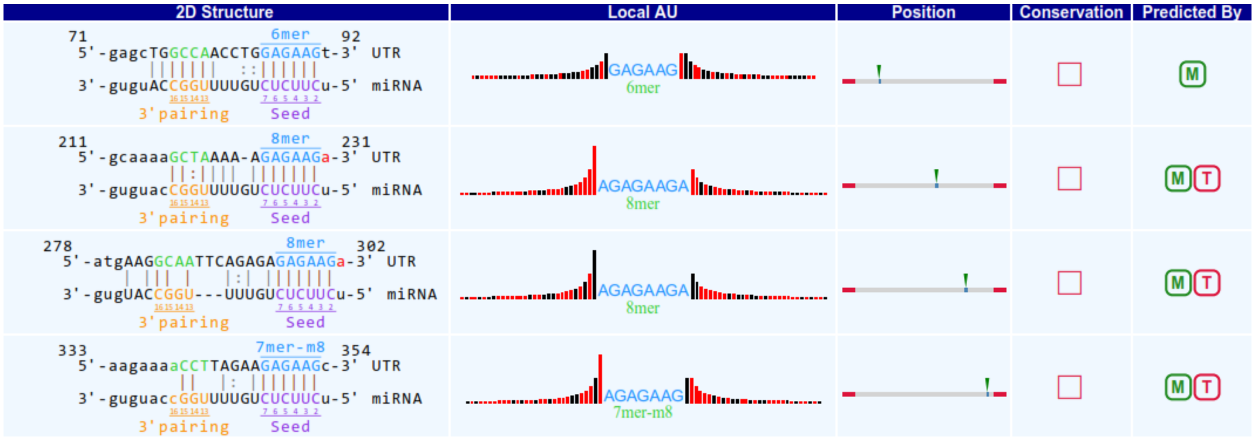

Figure 6: The binding interactions of hsa_circRNA_000479 with hsa-miR-942-5p response elements

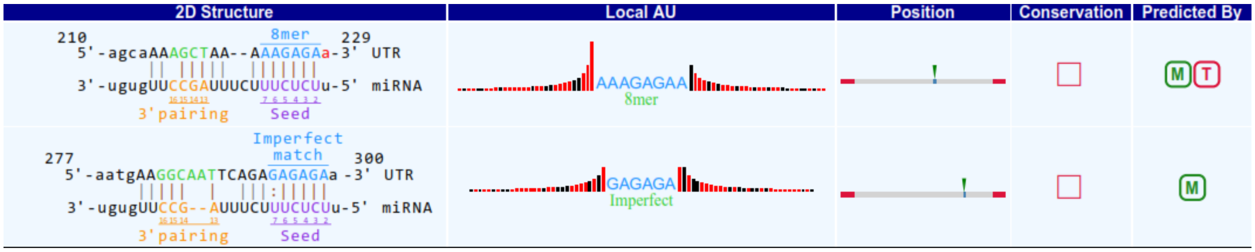

Figure 7: The binding interactions of hsa_circRNA_000479 with hsa-miR-4753-3p response elements

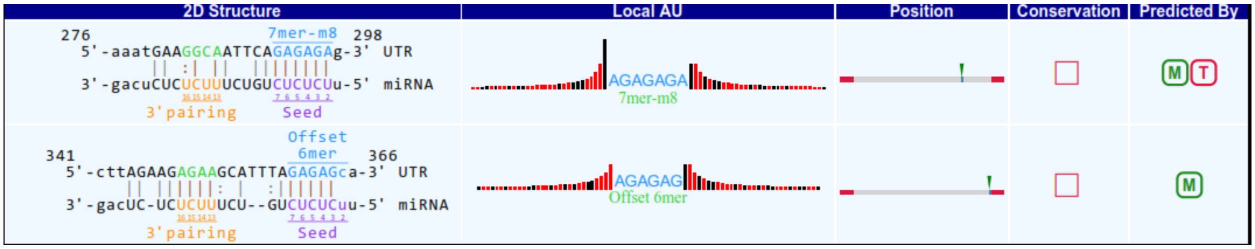

Figure 8: The binding interactions of hsa_circRNA_000479 with hsa-miR-6873-3p response element

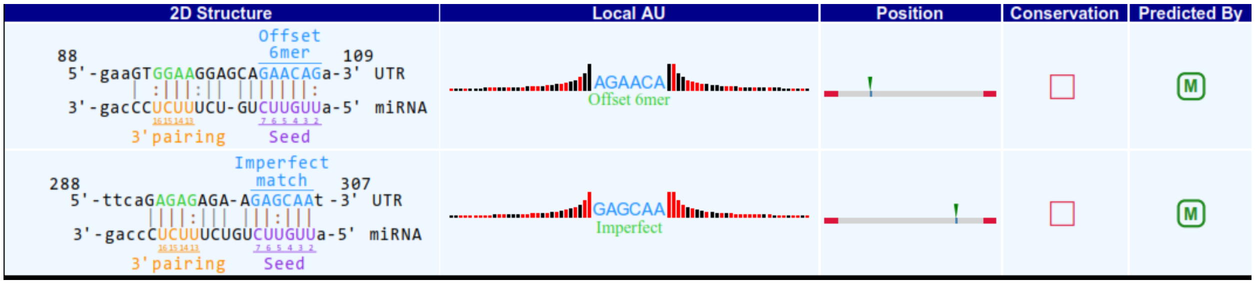

Figure 9: The binding interactions of hsa_circRNA_000479 with hsa-miR-6739-3p response element

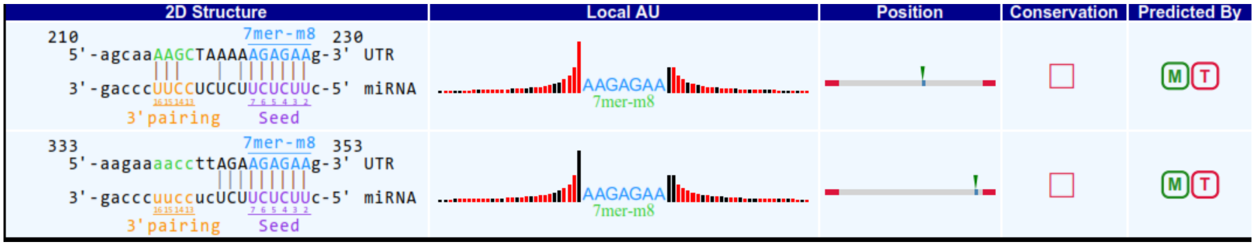

Figure 10: The binding interactions of hsa_circRNA_000479 with hsa-miR-6809-3p response element
